# Implementing and Assessing a Resident Diversity Council to Address the Need for a Diverse Clinical Workforce

**DOI:** 10.1001/jamanetworkopen.2022.38240

**Published:** 2022-10-24

**Authors:** Cody Gathers, Camila M. Mateo, Colin Sox

**Affiliations:** 1 Department of Anesthesiology and Critical Care Medicine, Children’s Hospital of Philadelphia, Philadelphia, Pennsylvania; 2 Division of General Pediatrics, Boston Children's Hospital, Boston, Massachusetts; 3 Harvard Medical School, Boston, Massachusetts; 4 Division of General Academic Pediatrics, Boston Medical Center, Boston, Massachusetts; 5 Boston University School of Medicine, Boston, Massachusetts

## Abstract

**Importance:**

Minoritized racial and ethnic groups remain underrepresented in medicine (UIM) compared with the general population. Although many residency programs want to train a diverse group of individuals, methods for implementation are not fully established.

**Objective:**

To describe the implementation and restructuring of the Boston Combined Residency Program (BCRP) Diversity Council and evaluate the association between restructuring the BCRP Diversity Council and the number of UIM interns.

**Design, Setting, and Participants:**

A retrospective cohort study was conducted on a large academic pediatric residency program at Boston Children’s Hospital and Boston Medical Center. Interns who matched in the BCRP from March 17, 2011, to March 18, 2021, were included. Interns who matched in an affiliated medicine-pediatrics residency were excluded because they are not universally exposed to the same recruitment efforts as individuals in the other BCRP tracks.

**Exposure:**

Because the BCRP Diversity Council was restructured in 2016, 2011-2016 was defined as the prerestructuring era and 2017-2021 as the postrestructuring era.

**Main Outcomes and Measures:**

The primary outcome was the proportion of interns who self-identified as UIM.

**Results:**

A total of 516 BCRP interns from 2011 to 2021 were included. A total of 62 individuals (12.0%) identified as racial and ethnic identities UIM (ie, American Indian/Alaska Native, Black/African American, Hispanic/Latino, and Native Hawaiian/Pacific Islander). During the 6-year prerestructuring Diversity Council era, 27 of the 275 BCRP interns (9.8%) were UIM; 35 of 241 BCRP interns (14.5%) were UIM during the 5-year restructured Diversity Council era (χ^2^ *P* = .10).

**Conclusions and Relevance:**

In this cohort study, the number of UIM interns was higher after the BCRP Diversity Council was restructured, although the difference was not statistically significant. As the magnitude of the Diversity Council’s influence is multidimensional, perhaps studying additional aspects would have better captured its impact. The BCRP Diversity Council has expanded innovative recruitment initiatives, supported efforts to improve the resident experience, and collaborated with the residency and institutional leadership to promote an inclusive and antiracist learning environment.

## Key Points

**Question:**

Is implementation of a residency diversity council associated with changes in the recruitment of interns who are underrepresented in medicine?

**Findings:**

This cohort study conducted from 2011 to 2021 included 516 interns. The proportion of interns who self-identified as underrepresented in medicine was higher after the Boston Combined Residency Program Diversity Council was restructured in 2016, but the difference was not statistically significant.

**Meaning:**

The implementation of a residency diversity council may increase the recruitment of interns underrepresented in medicine in a residency program.

## Teaser

This cohort study examines the implementation of a diversity council and changes noted in the number of residents underrepresented in medicine after restructuring of the council.

## Introduction

Minoritized racial and ethnic groups remain underrepresented in health care compared with the general population. The American Association of Medical Colleges currently defines individuals who are underrepresented in medicine (UIM) as “those racial and ethnic populations that are underrepresented in the medical profession relative to their numbers in the general population.”^1^ In 2018, UIM physicians made up 11% of the physician workforce,^2^ although minoritized racial and ethnic groups account for 33% of the US population.^3^ Many training programs prioritize the importance of increasing diversity in health care as an avenue to improve care for underserved populations and eliminate health care disparities.^4^ Increased patient satisfaction and decreased time to initiation of protease inhibitor therapy for patients who are HIV-positive have been associated with racially concordant patient-physician relationships.^5^ Evidence also suggests that UIM physicians and physicians who trained at medical schools with racial and ethnic diversity may have a higher intention to practice in underserved communities and treat a greater proportion of minoritized racial and ethnic groups, patients on Medicaid, and uninsured patients.^6,7^ Moreover, increasing diversity in medicine may further assist in eliminating the effects of racism and implicit bias on patient care.^8^

To further address the need for a diverse clinician workforce, the American Association of Medical Colleges instituted a Call to Action focused on the publication of data and allocation of resources to implement programs that further recruit UIM physicians.^9^ Although many efforts have focused on recruiting historically marginalized groups to medicine, only some have established official working groups to recruit, educate, and retain a diverse clinical workforce.^10^ In an effort to improve the recruitment of UIM physicians to the Boston Combined Residency Program (BCRP), the BCRP Diversity Council leveraged intersectionality, integrated diversity leadership efforts into program leadership, and created collaborative leadership models between trainees and faculty. The aims of this study were to describe the goals, formation, and restructuring of the BCRP Diversity Council and evaluate the association between its restructuring and the number of UIM interns in the residency program.

### Formation and Restructuring of the BCRP Diversity Council

Although efforts existed for diversity and inclusion within the residency program, including a holistic review application process, UIM applicant recruitment dinners, and institutional participation in Student National Medical Association and Latino Medical Student Association conferences, these efforts lacked a clear structure and residents were minimally involved in the planning and execution of these efforts. Residents of diverse backgrounds who were passionate about diversity and inclusion and recognized its importance in health care and resident education created a Diversity Council in 2014 to help create community among current residents and improve diversity recruitment to the BCRP. However, the Diversity Council was not directly integrated into the residency organizational or leadership structure. In 2016, chief residents led a collaborative working group of administrators, residents, alumni, and faculty leadership within the BCRP to coordinate and implement diversity and inclusion efforts.

This working group recognized an innovative opportunity to center resident voices in diversity and inclusion efforts by restructuring and expanding the BCRP Diversity Council. These innovations included centering the intersectionality of diversity, the seamless integration of program leadership into its structure, and a creative organization that included specific working groups in conjunction with a shared leadership model that was resident led and faculty supported.^11^

Before the restructuring of the BCRP Diversity Council, diversity programming focused exclusively on traditionally underrepresented racial and ethnic groups in medicine. The BCRP Diversity Council centered themes of inclusivity and intersectionality as guiding principles in the restructuring process. This process included broadening the BCRP’s definition of diversity in recruitment to include those who identified as underrepresented based on race, ethnicity, sexual orientation, gender identity, socioeconomic status, and/or disability and allowing applicants to self-identify during the recruitment process to participate in diversity recruitment events. The BCRP expanded Diversity Council membership to include residents who identified as underrepresented in any of the ways listed above as well as residents who did not identify as underrepresented but were passionate about diversity and inclusion efforts in the BCRP with a desire to participate in this important work.

Next, the BCRP Diversity Council leadership was integrated into program leadership through the establishment of a diversity chief resident who served as a liaison and leader of both the Diversity Council and the BCRP. The integration of the BCRP Diversity Council into program leadership led to greater coordination, communication, and implementation of diversity efforts. Through the integration of the BCRP Diversity Council into program leadership, advocating for needed resources was more streamlined, contributing to successful advocacy for more funding toward BCRP Diversity Council initiatives, including increased budgets for UIM recruitment dinners provided before every interview day and professional development sessions for current residents. In addition, the BCRP Diversity Council used a creative organizing strategy to both help prioritize program goals and ensure a shared leadership model.

The BCRP Diversity Council was divided into 3 working groups consisting of recruitment, community-building, and education; each was tasked with different strategic goals to ensure diversity and inclusion were being prioritized throughout the program. Each working group was led by 1 to 2 resident leaders who were supported by a faculty member. This structure facilitates the development of resident leadership by prioritizing resident ideas and perspectives as initiatives are designed and implemented. Faculty support ensures appropriate mentorship, provides continuity as residents graduate, and can prevent residents from being overburdened in these leadership roles.

### Current Model of the BCRP Diversity Council

**Figure 1.**
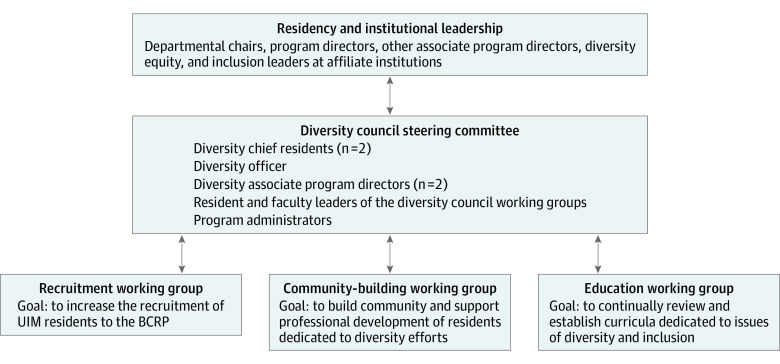
Structure of the Boston Combined Residency Program (BCRP) Diversity Council UIM indicates underrepresented in medicine.

The current organization of the BCRP Diversity Council provides a sustainable and effective residency-wide structure to promote diversity and inclusion in the BCRP (Figure 1). As mentioned above, the BCRP Diversity Council is divided into 3 distinct but synergistic working groups: recruitment, community-building, and education. Each working group is made up of resident leaders and at least 1 faculty leader. Resident members of the BCRP Diversity Council are free to join any working group. The diversity chief resident creates a direct connection between BCRP Diversity Council initiatives and program leadership. Since the restructuring of the BCRP Diversity Council in 2016, this role has expanded to include 2 diversity chief residents each year, as well as new roles for faculty including a faculty diversity officer and associate program directors with diversity initiatives as a part of their role in the BCRP. This group works closely with the resident and faculty leaders from each working group. Together, the BCRP Diversity Council works to guide, implement, and coordinate diversity efforts from each working group throughout the residency program and collaborate with institutional affiliates, such as our hospitals and medical schools, on diversity, equity, and inclusion (DEI) initiatives.

The goal of the recruitment working group is to increase the number of UIM residents in the residency program. Since its establishment in 2016, the recruitment working group has implemented a structured advisory relationship program between current residents and intern applicants, organized recruitment dinners for applicants from diverse backgrounds, increased institutional funding allocated to BCRP representatives to ensure a larger and more reliable presence at Student National Medical Association and Latino Medical Student Association regional and national conferences, and implemented a fully funded second-look event for UIM applicants who interview with the residency program. The community-building working group was created to maintain a strong community of support and mentorship for residents within the BCRP. It does so by working to establish stronger faculty-resident mentorship and professional development for current residents through structured professional development sessions with faculty. Professional development sessions serve as an opportunity for residents to meet UIM faculty and learn about their career trajectory. The goal of the education working group is to continually review our current curricula and when indicated, work to establish DEI-related curricula to address identified gaps through curriculum mapping and resident surveys.^12^

In summary, this diversity council model offers a unified and comprehensive approach to achieve institutional change around diversity and inclusion. The 3 working groups implement interventions to address the shortage of diversity in medicine while also working to highlight and amplify the benefits of improved diversity within the health care workforce.

## Methods

We performed a retrospective cohort study over an 11-year period in the BCRP to assess the association between the restructuring of the BCRP Diversity Council and the number of UIM BCRP interns. This study followed the Strengthening the Reporting of Observational Studies in Epidemiology (STROBE) reporting guideline for observational studies.^13^ The Boston Medical Center and Boston Children’s Hospital institutional review boards determined that this study qualifies for an exemption determination under the policies and procedures of the Human Research Protection Program.

### Study Population, Variables, and Outcomes

The study population included individuals who matched into any of the following tracks of the BCRP from March 17, 2011, to March 18, 2021: categorical, leadership in equity and advocacy (formerly urban health and advocacy), child neurology, neurodevelopmental disabilities, pediatric anesthesia, and pediatrics-medical genetics. Individuals who matched in the BCRP medicine-pediatrics residency were excluded because these they are not universally exposed to the same recruitment efforts as used by the other BCRP tracks. The primary independent variable was whether an intern self-identified as UIM; applying the American Association of Medical Colleges 2004 definition, UIM groups included American Indian/Alaska Native, Black/African American, Hispanic/Latino, and Native Hawaiian/Pacific Islander.^1^ We categorized interns who matched into the BCRP from 2011 to 2016 as training in the prerestructuring of the BCRP Diversity Council era, and those who matched from 2017 to 2021 were categorized as experiencing the postrestructuring of the BCRP Diversity Council era. In accordance with the original institutional review board, the BCRP Resident Research Committee, and program directors, we did not collect data on the sexual identification of study participants to maintain anonymity due to the sensitive nature of reporting demographic information on underrepresented populations.

### Statistical Analysis

The statistical associations between the BCRP Diversity Council restructuring eras and the number of UIM interns were assessed with χ^2^ tests using Stata, version SE 16.1 (StataCorp LLC). A 2-sided hypothesis was used, and *P* < .05 was considered to be statistically significant.

## Results

**Figure 2.**
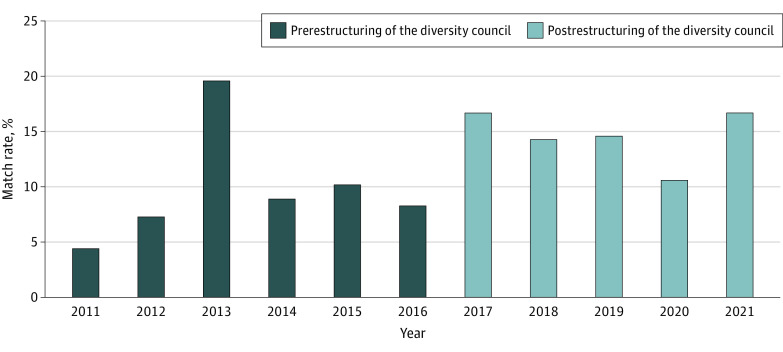
Annual Underrepresented in Medicine Match Rate

A total of 516 interns matched into the BCRP between 2011 and 2021. A total of 62 individuals (12.0%) identified as racial and ethnic identities UIM (ie, American Indian/Alaska Native, Black/African American, Hispanic/Latino, and Native Hawaiian/Pacific Islander). During the study period, the annual UIM match rates (median, 10.4%; range, 4.4%-19.6%) for each of the 11 study years are presented in Figure 2 and did not significantly differ by year (overall χ^2^ *P* = .46). During the 6-year prerestructuring BCRP Diversity Council era, 27 UIM and 248 non-UIM applicants matched in the BCRP, while during the 5-year restructured BCRP Diversity Council era, 35 UIM and 206 non-UIM applicants matched in the BCRP. The prerestructuring BCRP Diversity Council era UIM match rate (9.8% of interns were UIM) did not significantly differ from this rate during the postrestructuring BCRP Diversity Council era (14.5%) (χ^2^ *P* = .10).

## Discussion

The structure of the BCRP Diversity Council is an innovative program that integrates 3 resident-led and faculty-supported working groups to promote diversity and inclusion within a residency program. Although the proportion of interns who self-identified as UIM was higher after the restructuring of the BCRP Diversity Council compared with before restructuring, this was not a statistically significant difference. The 2013 intern class in the prerestructuring cohort appears to be an outlier with a much higher recruitment of UIM interns than all other years in that era—an imbalance that may have limited our analyses. We have no clear explanation for the higher number of UIM interns in 2013, so presume it was due to random variation. It will be important to continue assessing UIM recruitment and the strength of this association over time. The current model of the BCRP Diversity Council bolsters recruitment efforts for UIM applicants, as the recruitment working group has enhanced initiatives supporting UIM recruitment. It will be important to consider bolstered efforts for the community-building working group and institutional support for leadership in evaluating solutions to UIM retainment at Boston Children’s Hospital or Boston Medical Center. The association between the educational working group and DEI-related education of BCRP residents and faculty was not evaluated.

In addition, the magnitude of the BCRP Diversity Council’s influence is difficult to measure and should not be undervalued. The BCRP Diversity Council has been instrumental in transforming the conversation of diversity and inclusion within the residency program. Purposeful prioritization of diversity and inclusion through specific diversity-related recruitment programing, institutional support through resource allocation, and increased visibility of UIM role models has been shown to be important for UIM recruitment.^14^ Consequently, the BCRP placed an intentional emphasis on UIM recruitment in the application and interview process reflecting prioritization of diversity within the residency program. Although there were no formal interventions implemented in the applicant interview or ranking process specifically, the importance of increased diversity in the selection of BCRP interns was a critical priority for those involved in the intern selection process.

The BCRP Diversity Council model may be applicable to other training programs. Before the restructuring of the BCRP Diversity Council, the group had little ability to implement changes and no leadership role in diversity efforts throughout the residency. After its restructuring, the BCRP Diversity Council was better able to prioritize resident leadership and community-building while also allowing for more streamlined implementation of innovative programing by integrating BCRP Diversity Council leadership with BCRP leadership. Other training programs can consider establishing structures similar to the BCRP Diversity Council in their program to better integrate resident-led diversity and inclusion priorities into their residency diversity and inclusion efforts. Moreover, selecting a chief resident who coordinates initiatives between the diversity council and program leadership can be critical for its success. The integration of program leadership in the structure of the diversity council is paramount to ensuring initiatives are supported and funded. In addition, supporting the faculty in these endeavors is equally important. At the time of restructuring the BCRP Diversity Council, the faculty who were involved volunteered their time, and there was a concern that residents and chief residents could be overburdened by coordinating the BCRP Diversity Council and its program-wide efforts. Recently, there has been a broad effort to address this issue within the residency by establishing protected time for 2 associate program directors to support BCRP Diversity Council efforts.^15^

### Future Directions

Future steps for the BCRP Diversity Council will include continued support and innovation of recruitment initiatives and evaluating the community-building working group’s association with the resident experience. In addition, there will be continued communication and collaboration between the BCRP Diversity Council, residency, and institutional leadership on ways to create a more inclusive and antiracist learning environment. The BCRP Diversity Council will formally evaluate applicants, residents, and faculty on diversity engagement. In addition, the BCRP Diversity Council will integrate formal DEI training in resident education and continue to develop and evaluate formal curricula that address topics affecting diversity in health care.

### Limitations

This study has limitations. The evaluation of the association between restructuring the BCRP Diversity Council and the number of UIM interns is limited by many factors. First, this was a nonrandomized single-center study with a moderate sample size. The evaluation of BCRP UIM recruitment does not account for other unmeasured changes within the residency program and institutions that may have affected diversity and inclusion. For example, it is difficult to ascertain whether the review of UIM applicants changed meaningfully during the study period. In addition, the impact of the BCRP Diversity Council may not be best measured by UIM recruitment alone; perhaps surveying residents and faculty regarding the current climate of diversity engagement (potentially using the validated American Association of Medical Colleges Diversity Engagement Survey^16^) may enhance understanding the outcomes of the BCRP Diversity Council in the learning environment. Surveying UIM applicants about their experiences during their interview day may provide insight into the role the BCRP Diversity Council plays in their evaluation of the program. Lack of formal DEI training for residents in the BCRP Diversity Council may limit its efforts in UIM recruitment. Given the current feasibility of DEI training, the BCRP Diversity Council will strongly consider advocating for the integration of formal DEI training to be implemented in resident education.

## Conclusions

The structure and implementation of the BCRP Diversity Council may provide a useful model for bolstering recruitment, community-building, and education efforts related to DEI in a residency program. An expanded definition of diversity that includes race, ethnicity, sexual orientation, gender identity, socioeconomic status, and/or disability, creating a brave space for people with intersecting identities, is a potential avenue for residency programs to foster a diverse and inclusive environment for its trainees.
